# Reduced categorical congruence of cognitive and affective empathy in persons with psychotic disorders

**DOI:** 10.1038/s41598-025-34560-9

**Published:** 2026-01-14

**Authors:** S. Morini, L. Hölz, A-C. S. Kimmig, B. Derntl, D. Wildgruber

**Affiliations:** 1 Department of Psychiatry and Psychotherapy, University of Tübingen, Calwerstaße 14, 72076 Tübingen, Germany; 2 German Center for Mental Health (DZPG), Partner Site Tübingen, Tübingen, Germany

**Keywords:** Compassion, Emotion, Feeling, Schizophrenia, Textual empathy test, Theory of mind, Neuroscience, Psychology, Psychology

## Abstract

Empathy encompasses the ability to recognize the emotional states of others (cognitive empathy), adjust one’s own feelings accordingly (affective empathy), and develop the motivation to provide support (compassionate empathy). Emotional categorical congruence describes the similarity between the emotions we attribute to another person or experience ourselves in response to their situation and that person’s original emotional state. Some authors argue that categorical congruence is an essential feature of empathy, while others emphasize that similarity regarding the hedonic quality of the respective emotional states is sufficient. Several studies have reported empathic deficits in patients with psychotic disorders. Here, the categorical congruence of empathic responses was examined in 21 individuals with psychotic disorders and 21 healthy controls. After reading brief descriptions of emotional situations, participants categorized both the emotional state of the person described in the scenario and their own emotional state, as the other person experiences the situation, using five positive and five negative emotion categories. Additionally, participants rated their motivation and ability to support the other person in the given situation. Patients with psychotic disorders showed reduced categorical agreement between cognitive and affective empathy, while both aspects of compassionate empathy remained unaffected.

**Supplementary Information:**

The online version contains supplementary material available at 10.1038/s41598-025-34560-9.

## Introduction

Among many other definitions, empathy has been described as “The ability to recognize other’s affective states and share the emotional experience associated to these affective states while maintaining a self-other distinction”^1^. From this definition, it is evident that empathy is inherently an interpersonal process, occurring between the empathizing individual and the person whose affective state is being shared or inferred^2^. This capacity to communicate, understand feelings, and perceive emotional states is critical prerequisite for a successful social interaction, and, thus, a vital skill in our society^3^.

Empathy has, over time, been deconstructed into two main components: (1) *cognitive empathy*, i.e. the ability to recognize and understand how others are feeling^4^, and (2) *affective empathy*, i.e. the emotional reactions a person experiences in response to what another person feels. In addition to these two components, Paul Ekman introduced, within his working definition of empathy^5^ a third component called (3) *compassionate empathy*, defined as the desire to help and support another person in dealing with their situation and emotions^6^. The success of compassionate empathic interventions, however, depends not only on the intensity of the motivation to provide support, but also on the effectiveness of the available interactional skills used to aid the other person’s emotion regulation. Accordingly, compassionate empathy can be differentiated into the aspects of motivation and ability to support others^7^.

Another notable sub-facet of empathy is the degree to which the observer’s cognitive or affective state during empathic processes matches the observed person’s affective state at the level of emotional categories. In other words, whether the empathic emotion assumed (for cognitive empathy) or felt (for affective empathy) by the observer when the other person is experiencing a situation and the actual emotional state of the observed individual belong to the same emotional category. This element is defined as (emotional) categorical congruence. There are differing opinions on whether categorical congruence should be included in the definition of empathy^8^. For instance, some authors, as reported by Bonfils et al.^9^, emphasize that affective empathy must indeed match the emotional state of the person one is empathizing with (i.e., include categorical congruence in the definition of affective empathy), while others underline more the importance of similar emotional valence (i.e., the hedonic quality of the emotion, ranging from negative to positive) of the empathic reaction^10^. Without taking a position in this debate, but finding the discussion itself intriguing, the present study chose to investigate in depth the components of categorical congruence and its manifestation in patients with psychotic disorders and in healthy controls.

Patients diagnosed with psychotic disorders often exhibit compromised social functioning, which is a key feature of the disorder^11^. It has been reported that deficits in social interaction tend to worsen over the course of the disease, with a negative impact on relapse frequency (as reviewed by Pinkham et al.^12^. The significance of empathy in fostering healthy and protective social interactions has been already well established. It is therefore of great importance to understand the facets of deficits in individual empathy components in patients with psychotic disorders, in order to better assess their impact on social functioning and quality of life, and to develop effective therapeutic interventions that can help to reduce these impairments^13^.

Human beings express emotions both verbally and through nonverbal visual and auditory cues (such as facial muscle contractions or variations in vocal tone and volume). Several studies have examined the perception of nonverbal emotional cues in patients with schizophrenia. Numerous investigations have reported impairments in recognition of emotional facial expressions^3,14–17^, emotional prosody^18,19^ as well as in tasks combining facial expressions and tone of speech^20,21^. Meta-analyses of these studies have consistently confirmed the presence of deficits in identifying and categorizing nonverbal emotional cues in patients with schizophrenia^22–24^. However, all the tests used in the aforementioned studies provided emotional nonverbal cues (e.g., visual and auditory stimuli) to the participants. Deficits at the perceptual level of these cues would hinder the independent investigation of deficits in specific components of empathic processing such as cognitive empathy, affective empathy, and compassionate empathy. Additional evidence has demonstrated cognitive empathy deficits in patients with psychotic disorders in terms of intensity using the self-reported IRI-Perspective-Taking subscale^25^ and QCAE^26^, which is free of nonverbal cues. This has been confirmed by a meta-analysis^27^ and subsequent studies^28–30^. With regard to affective empathy, a comprehensive meta-analysis^9^ - including primarily self-reported measures such as the IRI-Empathic Concern subscale^25^, but also performance-based measures including pictures as nonverbal cues^16,31^ - found evidence of deficits in this component as well. In contrast, there is, to our knowledge, no clear evidence in the current literature regarding whether compassionate empathy is impaired in patients affected by psychotic disorders. In these individuals, a tendency to experience so-called “fear of compassion” (e.g., defensive emotions and avoidance when giving or receiving compassion) has been reported^32^.

Unlike the tools used in previous studies, the task employed in this work, the Textual Empathy Test (TET; originally “Tübinger Empathy Test“^33,34^ is both performance-based and independent of possible impairments in the perception of nonverbal emotional cues, and it allows to assess the components of empathy separately. The TET uses textual descriptions of emotional situations to assess cognitive, affective, and compassionate empathy toward a target person in different situations. It has previously been employed to evaluate empathy in individuals with autism spectrum disorder^35^ and women with different hormonal states^33,34^. It includes an equal number of positive and negative emotional scenarios, with the original version including a friend, a foe or a stranger to investigate the influence of closeness on empathy components. Regarding the target person, the version used in this study focuses solely on scenarios involving unknown persons, to keep the total length to a reasonable duration of around 35 min, even with the addition of categorical congruence questions.

We predicted that patients with psychotic disorders would exhibit reduced categorical congruence in both cognitive and affective empathy in the TET compared to healthy controls, analogous to their deficit in emotion-recognition and performance-based tasks^16,22–24,31^. In addition to testing these hypotheses, the present study aimed to address the following exploratory questions: whether patients with psychotic disorders also show disturbances in the motivation and ability aspects of compassionate empathy, and to what extent disturbances in empathic responses are influenced by specific components of empathy (i.e., cognitive vs. affective empathy), emotional valence (positive vs. negative emotions) or specific emotional categories (e.g., shame, sexual arousal).

## Results

In total, 42 participants (21 patients with psychotic disorder and 21 healthy controls) were enrolled in this study. The patient and control groups were matched with regards to sex and age (see Table 1), but differed significantly in IQ values.

**Table 1:** Comparison of demographic characteristics and IQ between patients with psychotic disorders and the healthy control groups. Data presented in form: average ± standard deviation; range. IQ: intelligence Quotient.

	Patients (*n* = 21)	Controls (*n* = 21)	t / χ²	*p*
Gender			0	1
Female	6	6		
Male	15	15		
Diverse	0	0		
Age	36.6 ± 11.6; range 21–56 years	35.4 ± 12.4; range 23–65 years	0.32	0.750
IQ	101.8 ± 12.7; range 70–130	111.9 ± 16.1; range 91–143	− 2.27	0.029

### Categorical congruence in cognitive and affective empathy

**Fig. 1:**
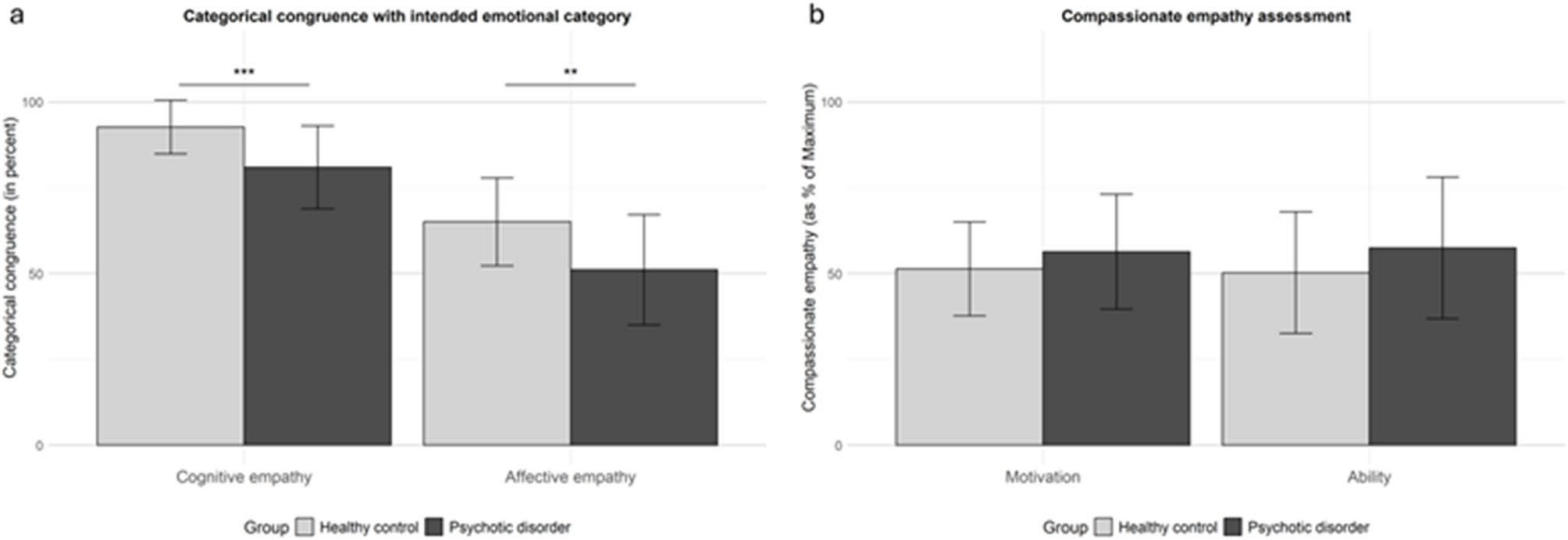
Difference in categorical congruence and compassionate empathy levels among patients and controls. The error bars indicate the respective standard deviations. (**a**) The columns represent the mean categorical congruence (in percent) in emotional situations while rating how the other person feels experiencing the current situation (cognitive empathy) and how the participant feels her-/himself when the other person experiences the situation (affective empathy). (**b**) The columns represent self-estimated values of compassionate empathy for the motivation and ability aspects. ***p < 0.001, **p < 0.01.

Regarding the main hypotheses, two *t*-tests for independent samples showed that patients with psychotic disorders exhibited significantly lower categorical congruence than the control group with respect to both cognitive (Patients: Mean = 0.81, SD = 0.12; Controls: Mean = 0.93, SD = 0.08; t(40) = − 3.75; *p* < 0.001, with a large effect size: Cohen’s d = − 1.16) and affective empathy (Patients: Mean = 0.51, SD = 0.16; Controls: Mean = 0.65, SD = 0.13; t(40) = − 3.12; *p* = 0.002, with a large effect size: Cohen’s d = − 0.96) - see Fig. 1a.

In order to address the exploratory questions, a repeated measures ANCOVA was calculated, using the empathy component (cognitive or affective) and the emotional valence (positive or negative) as within-subject factors, the participant’s group (patient or control) as between-subject factor, and the IQ as covariate. This ANCOVA showed a significant main effect for group (F(1,40) = 13.13; *p* < 0.001), with lower congruence in patients. No significant main effects were found for empathy component or emotional valence and no significant interactions between factors were detected.

## Compassionate empathy levels

**Fig. 2:**
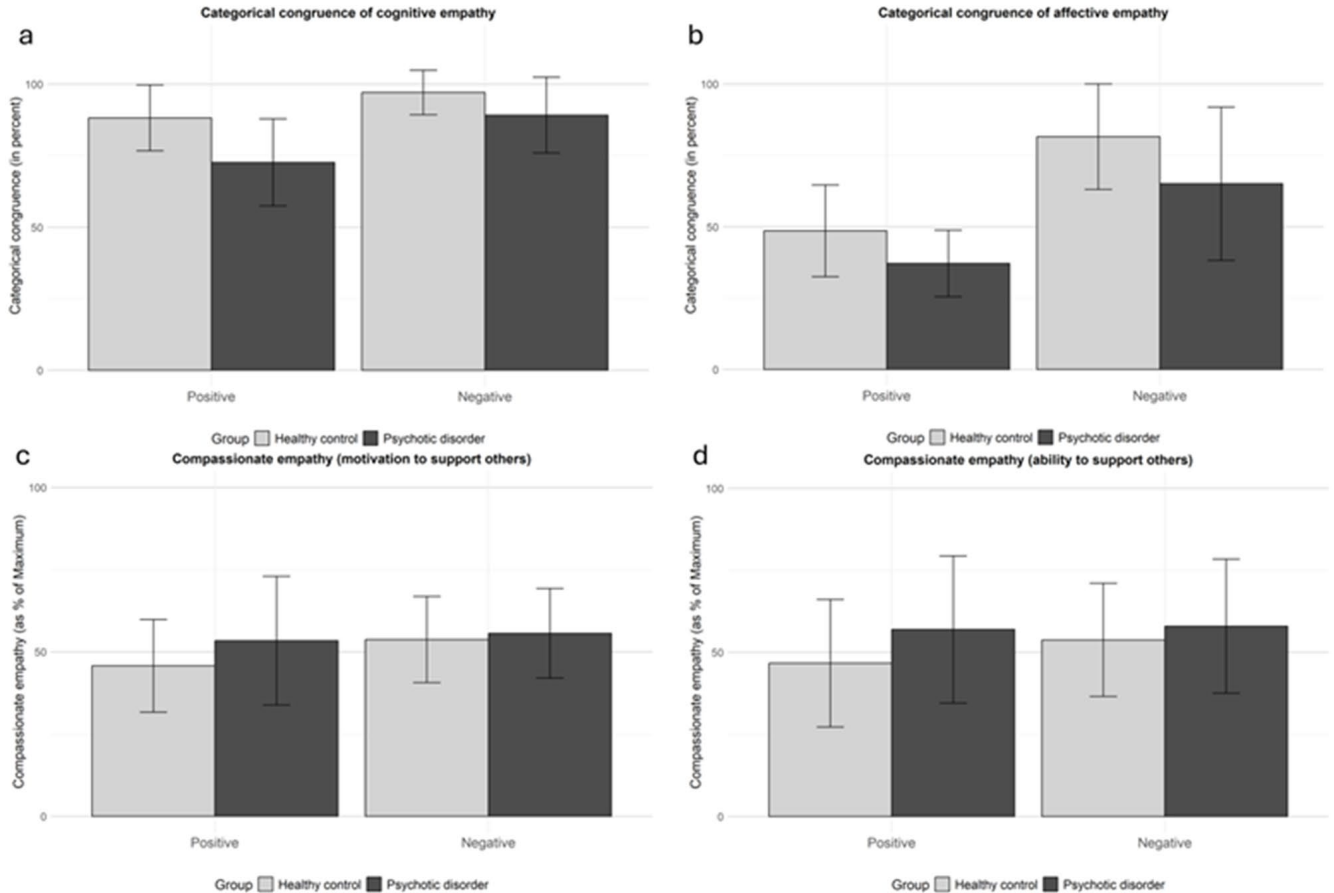
Difference in categorical congruence and compassionate empathy levels for positive and negative emotions between patients and controls. The error bars indicate the respective standard deviations. (**a**, **b**) The columns represent the mean categorical congruence (in percent) in emotional situations while rating how the other person feels experiencing the current situation (cognitive empathy, **a**) and how the participant feels her-/himself when the other person experiences the situation (affective empathy, b). (**c**, **d**) The columns represent self-estimated values of compassionate empathy for the motivation (**c**) and ability (**d**) aspects. *p < 0.05, ***p < 0.001.

In order to further investigate exploratory questions, two separate ANCOVAs (with IQ as covariate) were calculated to evaluate the influence of emotional valence on the motivation and ability aspect of compassionate empathy and revealed no significant main effects of group or valence and no significant interactions between these factors for the motivation as well as the ability aspect (see Fig. 2c, d).

## Emotion-specific differences in categorical congruence rates and compassionate empathy levels

**Fig. 3:**
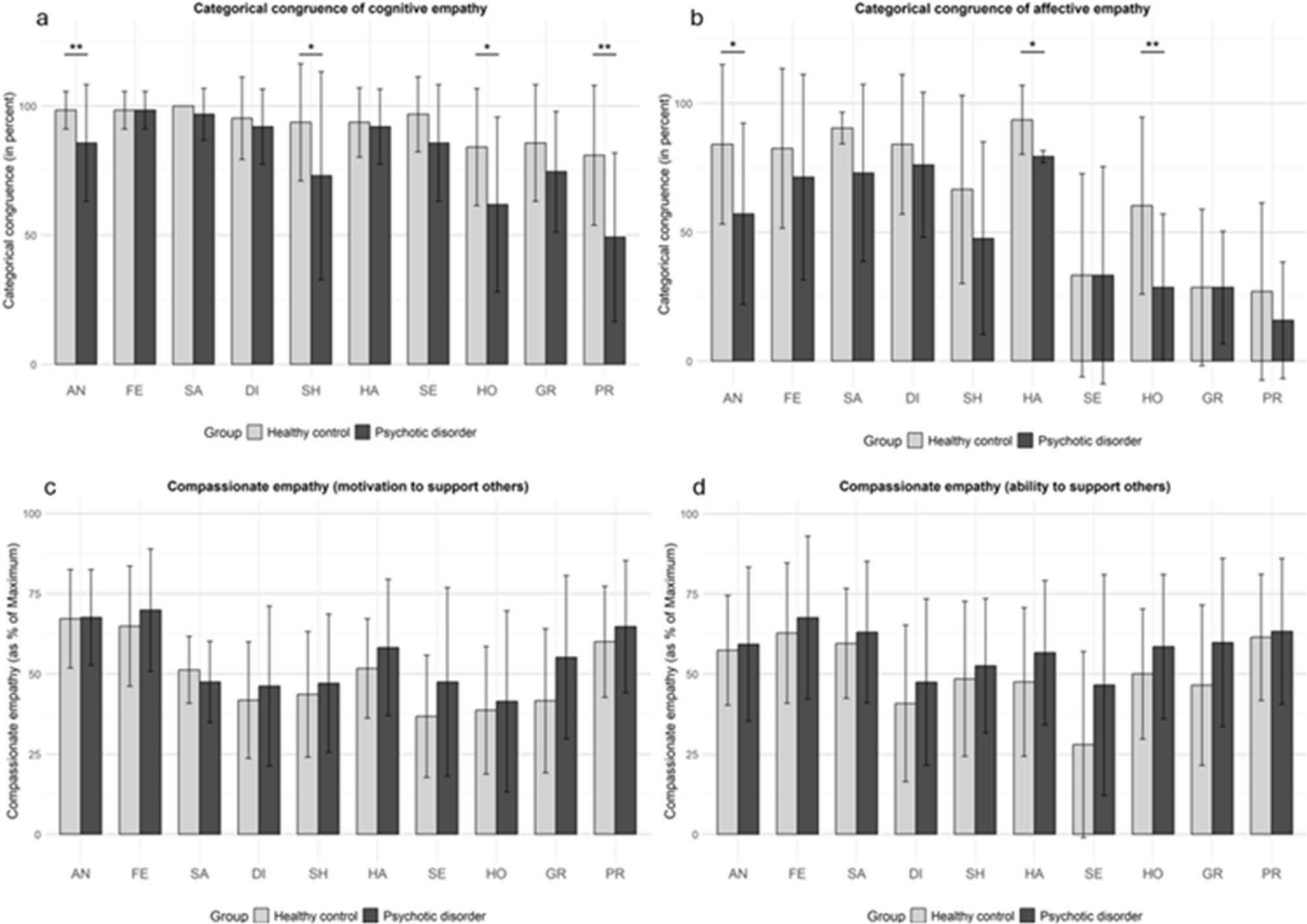
Emotion-specific differences in categorical congruence levels for cognitive (**a**) and affective (**b**) empathy and in levels of motivation (**c**) and ability (**d**) aspects of compassionate empathy between patients and controls. The error bars indicate the respective standard deviations. (**a**, **b**) The columns represent the mean categorical congruence (in percent) with the intended emotion while rating how the other person feels experiencing the current situation (cognitive empathy, **a**) and how the participant feels her-/himself when the other person experiences the situation (affective empathy, **b**). (**c**, **d**) The columns represent self-estimated values of compassionate empathy for the motivation (**c**) and ability (**d**) aspects. On the X-axis: AN: anger, FE: fear, SA: sadness, DI: disgust, SH: shame, HA: happiness, SE: sexual arousal, HO: hope, GR: gratitude, PR: pride. *p < 0.05, **p < 0.01.

Independent samples *t*-tests were performed to investigate emotion-specific differences in categorical congruence rates between patients and controls across both cognitive and affective empathy, as well as the motivational and ability components of compassionate empathy (Fig. 3). The statistical analysis revealed significantly lower congruence rates in patients compared to controls for the emotions anger, shame, hope, and pride in cognitive empathy, and for anger, happiness, and hope regarding affective empathy (see Supplementary material, Tables S3 and S4 and Fig. S3).

## Correlation of categorical congruence and compassionate empathy levels with IQ

Categorical congruence rates of cognitive empathy showed significantly positive correlations with IQ as measured by the German Multiple-Choice Vocabulary Intelligence Test (Spearman’s rho = 0.370, *p* = 0.016). However, when controlling for IQ as a covariate in a univariate ANCOVA, the main effect of group remained significant (F(1,39) = 9.96; *p* = 0.003, with a large effect size: _p_η^2^ = 0.20). Categorical congruence rates of affective empathy and compassionate empathy levels for the motivational as well as the ability aspect did not show significant correlation with IQ.

## Discussion

The aim of this study was to investigate the characteristics of empathy in patients with schizophrenia compared to healthy controls. For this purpose, an adapted version of the TET, a tool designed to assess empathy through textual descriptions of emotional scenarios, was used. The TET presents an equal number of positive and negative emotional scenarios (items representing five positive and five negative emotions). The version of the test used in this study was modified in two ways. First, categorical congruence questions were included for both cognitive and affective empathy, in order to investigate this feature of empathy, which until now has mostly been studied using nonverbal emotional cues. Second, two additional questions were added for each emotional item to explore differences in compassionate empathy between patients and controls, focusing specifically on a self-assessment of the motivation and the ability to emotionally support the other person, which have not been thoroughly investigated so far.

In line with previous studies that revealed deficits in both cognitive and affective empathy in schizophrenia^9,22–24,27^, categorical congruence was lower in patients compared to controls for both the cognitive and affective empathy components. Cognitive empathy correlated significantly with IQ, and lower IQ scores in the patient group partially explain the reduction in cognitive empathy observed in individuals with psychotic disorders. Affective and compassionate empathy, however, showed no significant correlation with IQ.

Previous studies observed deficits in perception and identification of nonverbal social cues in patients with psychotic disorders. Impairments in empathy tasks requiring recognition of nonverbal cues might therefore be partially explained by perceptual deficits. Since the TET did not provide nonverbal communicational signals, the observed impairments cannot be attributed to difficulties in decoding nonverbal social cues. With regard to cognitive empathy, the effect size obtained in this study (d = 1.16) was comparable to that reported in other performance-based studies, including meta-analyses on facial recognition task^22,23^ and recognition of emotional prosody^24^, and larger than that found for the IRI-PT scale (d = 0.66)^27^. In terms of affective empathy, the effect observed in this study was larger than that reported in meta-analyses of the IRI-EC scale alone (d = 0.21)^27^ or combined with other performance-based measures (g = 0.36)^9^. This effect size gap between the TET (which is at least partially performance-based) and self-reported measures such as the IRI scale is consistent with findings from studies showing that patients with psychotic disorders tend to overestimate their empathic abilities when compared to assessments by observers or family members^36,37^.

Furthermore, the present study explored whether patients exhibit lower levels of compassionate empathy compared to control subjects. The comparison of both the motivational and ability aspects of compassionate empathy did not reveal significant differences between the two groups. Thus, despite patients showing reduced ability to correctly identify others’ emotional states (cognitive empathy) and frequent incongruencies regarding the emotional category of affective empathy, they appeared to retain both the desire and the capacity to support others emotionally. Looking for related findings in the literature, studies investigating prosocial behaviour in schizophrenia offered some parallels, though often using different constructs. For example, Horat et al.^38^ using the Ultimatum Game^39^, a socioeconomic decision-making task reported that patients with schizophrenia made significantly more hyper-fair offers compared to controls. Research on altruism, a feature closely related to prosocial behaviour and described as behaviour intended to promote others’ well-being^40^, has shown that schizophrenia patients appear to have an impaired ability to deceive and manipulate others and, as a result, behave more altruistically^41^. In addition, genetic studies have identified variants of certain genes that are simultaneously associated with an increased risk of psychosis and a greater propensity for altruism. On the other hand, persons with psychotic disorders have been observed to exhibit increased rejecting responses when receiving compassion, as well as more cautious and reserved behaviour when providing support^32^. These defensive emotional reactions conceptualized as “fear of compassion” may be related to a reluctance to burden others when asking for or giving compassion. This, in turn, might be linked to past experiences of uncomfortable closeness or feelings of being influenced or controlled by others. The co-occurrence of an increased inclination toward altruism and a fear of compassion might contribute to reluctance in close social interactions, despite a general willingness to pursue altruistic goals. In contrast to affective empathy and compassionate empathy, altruism does also not necessarily require shared affect or emotional resonance^42^.

As an exploratory question, this study aimed to investigate the categorical congruence and compassionate empathy levels across different emotions. In terms of categorical congruence, certain emotions (particularly sexual arousal) exhibited a pattern of significant incongruencies in both cognitive and affective empathy (see Supplementary Material, Fig. S3). In contrast, for the motivational and ability aspects of compassionate empathy, no significant emotion-specific differences were found. This suggests that prosocial behaviour could be generally preserved across all emotions. Although compassionate empathy levels tended to be higher for negative compared to positive emotions, they did not differ significantly between patients and controls for any specific emotion.

In conclusion, the TET proved to be suitable for characterizing empathy in individuals affected by psychotic disorders, as it confirmed previous findings in the broader literature. However, there are some limitations associated with this study.

The first concerns the sample composition. Some measures, in which no (significant) differences were observed, might have yielded different results with a larger sample size. Additionally, the control group showed a high level of education and IQ values. Importantly, the observed group effect of reduced categorical congruence in cognitive empathy (which was positively correlated with IQ) remained significant even after controlling for IQ differences. Nevertheless, it would generally be preferable to match patients and controls on IQ and education level (in addition to gender and age) to minimize potential confounding effects.

Secondly, there are structural aspects of the TET that might have influenced the results obtained. The test contains a mixture of performance-based measures (primarily the questions regarding categorical congruence for cognitive empathy and, to some extent, affective empathy) and self-reported measures (categorical congruence of affective empathy and both aspects of compassionate empathy). To make the TET a fully performance-based task, additional physiological measures, like skin conductance, heart rate or functional MRI would need to be included, as has been done in previous studies^34,43–46^. Consequently, it is possible that participants misjudged or inaccurately reported the emotions they experienced or their intensity. Moreover, the assessment of emotional states in the TET is based solely on the perspective of an observer. In real-life situations, the possibility of bidirectional exchange of social signals between the persons involved can further increase the complexity of empathic processes^47,48^. Therefore, it is important to note that this interactional influence on empathic processes is not captured in the TET. Additionally, the included patients received antipsychotic treatment, which may have influenced the results. To our knowledge, no studies have yet investigated the effects of antipsychotics on empathic abilities. However, both positive and negative consequences are conceivable, as heterogeneous effects on cognitive abilities^49–51^ and negative symptoms^52^ have been observed, and sedation can occur as a side effect^53^.

Regarding its psychometric properties, the TET has been carefully developed and validated through two pilot studies with independent samples of healthy participants, which confirmed both the accuracy of the categorical assignment of items under forced-choice conditions and the intensity levels. They also showed that there was no significant difference in the average valence and arousal ratings between positive and negative emotions (Hölz et al., unpublished data). Moreover, the test has already proven useful in yielding significant results in previous studies^33–35^. Nevertheless, it should be noted that, unfortunately, test-retest measures for the TET have not yet been evaluated.

Furthermore, in the categorical congruence questions, only the option to choose among 10 emotions was given, with no possibility to select “none”. This limitation might have affected the results of categorical congruence, particularly for affective empathy. It is possible, in fact, that a participant might experience no emotional response while imagining the other person in the situation described. Therefore, adding an option for “none” should be considered in future iterations of the test.

As previously mentioned, this version of the TET does not include items in which a friend of the participant is experiencing a particular situation. This change was mainly intended to prevent the task from taking too long, which could lead to decreased attention, especially for patients with schizophrenia, who often have difficulty with sustained attention^54^. Additionally, not all participants may have friends, and the quality and intensity of friendships can vary, which could bias responses. However, this design choice likely contributed to lower measured levels of affective empathy (as has also been reported in patients with autism^35^ and compassionate empathy (compassionate responses to suffering strangers have been shown to engage different neural patterns compared to friends in similar situations^55,56^. Consequently, this may have led to an underestimation of the differences between the two groups.

One final shortcoming of the TET that could be a potential hint for further studies is its reliance on the assumption that every participant can evoke or imagine an emotional situation solely by reading a sentence. It might be that stronger empathic responses could be elicited using stimuli with a higher ecological validity, such as a text message from a real-life friend describing a situation they have experienced themselves, or a video depicting this person in a real-life scenario. Although these ideas are complex to implement in a study, they should nonetheless be acknowledged as potential directions for future research.

In conclusion, the present findings show lower levels of categorical congruence in cognitive and affective empathy, but not compassionate empathy in persons with psychotic disorders. Future work should additionally address the more complex challenge - beyond the scope of the present study - of translating self-reported measures of compassionate empathy into actual prosocial behaviour. Bridging this gap between experimental findings and real-life contexts could contribute to improving the quality of life of individuals affected by psychotic disorders.

### Methods

#### Participants

In total, 42 participants (21 patients with psychotic disorder and 21 healthy controls) were enrolled in this study over a period of approximately nine months. General inclusion criteria for both groups of this study were being 18–65 years of age and having very good knowledge of the German language (at least C1 level). Participants in the patient group had a DSM-5 diagnosis of either F20 (schizophrenia) or F25 (schizoaffective disorder). Additionally, all patients in this group were currently receiving antipsychotic medication and participating in psychotherapy. The diagnosis was made or revised by experienced medical staff at the University Clinic for Psychiatry and Psychotherapy in Tübingen.

Control group participants were required to have no psychiatric disorders and no first-degree relatives with a diagnosis of F20 or F25. Moreover, the structured diagnostic Mini-International Neuropsychiatric Interview (M.I.N.I.) did not identify any mental conditions in participants from the control group. Recruitment for the patient group was conducted directly at the University Hospital in Tübingen or its affiliated outpatient clinic. Matched control group participants have been recruited via convenience sampling.

## Procedure

All participants received a study description before commencing the experiment. The information was conveyed verbally and supported by pre-printed informational material, which was made available to every participant. After providing informed written consent, participants underwent the German Mehrfachwahl-Wortschatz-Intelligenztest (MWT-B) for IQ assessment. Following the completion of this test, they proceeded to the Textual Empathy Test (TET). The final task was the structured diagnostic Mini-International Neuropsychiatric Interview (M.I.N.I.) to screen for comorbidities. This step was intentionally scheduled at the end because it can be time-consuming for participants with numerous comorbidities, requiring significant concentration, which might have interfered with their ability to perform the TET. All participants received compensation (15 euro) for their participation. The study was approved on January 11, 2023 by the Ethics Committee at the Medical Faculty of the Eberhard Karls University and at the University Hospital of Tübingen and followed the ethical principles of the World Medical Association (Declaration of Helsinki).

### The textual empathy test (TET)

As the primary component of the experiment, all participants underwent the Textual Empathy Test, an empathy task adapted from previous studies^33–35^. The online questionnaire was generated using the software SoSci Survey^57^ and was completed via the website www.soscisurvey.de. The experiment was conducted in a quiet setting on a portable laptop with a stable internet connection. The duration of the test varied among participants, ranging from approximately 25 to 45 min.

This test involved the presentation of textual descriptions of potential real-life scenarios, each associated with a specific emotion chosen from a set of five positive emotions (happiness, pride, gratitude, hope, and sexual arousal) and five negative emotions (anger, sadness, fear, shame, and disgust). Sexual arousal was selected as a positive emotion in accordance with previous versions of the TET. For a balanced design of the TET five different positive and five different negative emotions were included. Within the basic emotion systems, distinct negative emotional categories are well established^58^ whereas happiness has been considered as the only positive emotion category. However, research during the last decades supported the distinction of further positive emotion categories^59^. Moreover, evaluations with the International Affective Picture System (IAPS^60^ and the International Affective Digitized Sounds (IADS^61^ demonstrated that pictures and sounds associated to sexual arousal consistently elicit the valence ratings comparable to those for other positive visual and acoustic stimuli and higher than those for neutral stimuli.

Each of the ten emotions had three scenarios assigned, differing from each other in intensity (light, medium and strong). All items included in the test had been validated in a preliminary study including 23 healthy participants, in which each item was presented from the participant’s own perspective and showed a correct categorical identification rate greater than 70% (See Supplementary Analysis and Table S2 for more detail). Moreover, the three intensity levels differed significantly from each other in terms of mean valence ratings (ranging from very unpleasant to very pleasant) and mean intensity ratings (the intensity of the specific emotion), whereas mean values of arousal and intensity did not differ between positive and negative emotions (unpublished data).

A detailed description of the complete test can be found in the Supplementary Material. Each situation was presented from the perspective of an unknown person whose gender matched the participant’s choice (female or male). Each distinct situation appeared on a different page, arranged in a randomized sequence newly generated by the software in every test run.

Participants were requested to read the presented scenario and imagine an unknown person experiencing the situation. Illustrative examples of the real-life scenarios presented in the test include the following (translated from the original German): “Ms./Mr. unknown is going to the concert of a famous musician” (associated with happiness), “Ms./Mr. Unknown is reading an erotic scene in a book” (associated with sexual arousal).

Each of the three components of empathy (i.e., cognitive and affective empathy - both analyzed in terms of categorical congruence - and compassionate empathy) was operationalized through four distinct questions. After imagining the situation, participants were requested to assign the emotion respectively:

- experienced by the unknown person (categorical congruence of cognitive empathy, i.e., the ability to identify another person’s emotional state), and.
- felt by themselves when the unknown person experiences the situation (categorical congruence of affective empathy, i.e., whether the participant’s own emotional reaction is of the same nature as the emotion experienced by the other).

to a category (happiness, sexual arousal, hope, gratitude, pride, anger, fear, sadness, disgust, or shame).

**Fig. 4:**
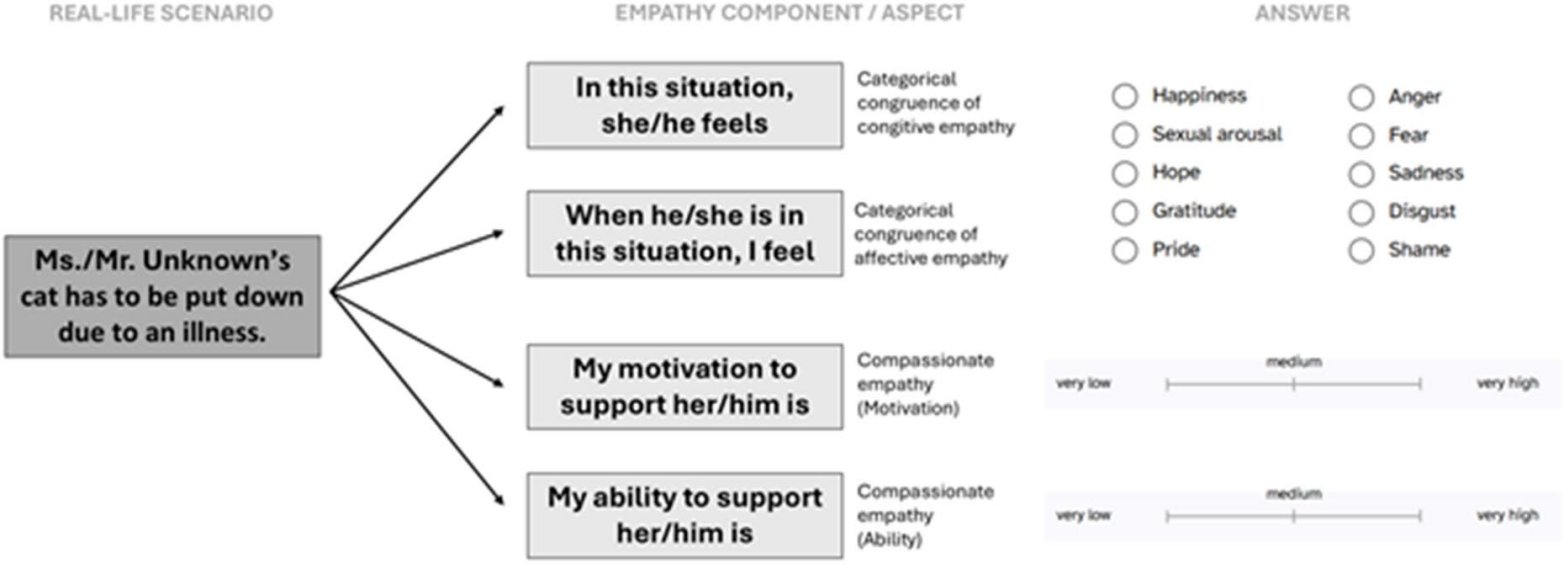
Task design. Participants were presented with different emotional scenarios involving an unknown person. They were instructed to imagine the pictured situation and then to infer: (1) how the unknown person feels in that situation (i.e., categorical congruence of cognitive empathy), (2) how they themselves feel when the unknown person experiences the situation (i.e., categorical congruence of affective empathy), and to rate (3) their motivation and (4) their ability to support the unknown person (i.e., compassionate empathy). For responses (1) and (2), participants had to select one of ten emotion categories. For (3) and (4), they had to indicate their response on a visual analogue scale. Additionally, on the same page, participants were requested to provide: (1) a self-estimation of the desire to emotionally support the unknown person, (compassionate empathy – motivation) and (2) a self-estimation of their ability to emotionally support the unknown person, (compassionate empathy – ability). The intensity of the compassionate empathy aspects had to be indicated on a visual analogue scale from “very low” to “very high”. See Fig. 4 for an overview of the collected variables.

Notably, in contrast to earlier original versions of the TET, the scene descriptions in the current version no longer vanish after a designated time interval. Instead, they persist on the screen remaining visible to participants until these actively click to proceed to the following situation and related questions.

### Data analysis

The statistical analyses were conducted using IBM SPSS Statistics for Macintosh, Version 29.0.1.1 (IBM, New York). Graphs were produced using the ggplot2 package (version 3.5.1) in R (version 4.3.2)^62^.

Calculation of the categorical congruence scores were performed on the tabular output of SoSci Survey using Python (version 3.9.13), with the pandas^63^ (version 1.4.4) and numpy^64^ (version 1.21.5) libraries in a Jupyter notebook environment^65^, and Microsoft Excel (Microsoft Corporation, Redmond, WA, USA). To calculate categorical congruence, the assignment of the emotion being experienced, respectively, by the unknown person (congruence of cognitive empathy) and by the participant themselves while imagining the unknown person experiencing the situation (congruence of affective empathy) was considered as congruent if it matched the intended emotional category that the scenario was designed to elicit (see Supplementary Material for more details). The average categorical congruence was calculated separately for each participant, emotion and empathy component (cognitive or affective). To evaluate the main hypotheses, differences between patients and controls in cognitive and affective empathy were assessed using *t*-tests for independent samples.

The two aspects of compassionate empathy (self-estimation of motivation and ability) were expressed for each individual as the mean of the values they provided on a visual analogue scale ranging from “very low” to “very high”.

To explore whether categorical congruence was greater for positive or negative emotions, average categorical congruence was calculated for each participant across all positive and negative emotions. In the ANCOVA calculation, the emotion’s valence (positive: happiness, sexual arousal, hope, gratitude, pride, or negative: anger, fear, sadness, disgust, shame) and the empathy component (cognitive or affective) were considered as within-subject factors, while the participant’s group (patient or control) was considered as between-subject factor and the IQ was used as covariate.

To investigate the levels of compassionate empathy for the two aspects, separate repeated measures ANCOVAs were performed for each, with the emotion’s valence (positive: happiness, sexual arousal, hope, gratitude, pride, or negative: anger, fear, sadness, disgust, shame) as the within-subject factor, the participant’s group (patient vs. control) as the between-subject factor, and the IQ as covariate.

Due to the robustness of ANCOVA to non-normally distributed data^66^, it was used to compare group means even though the dependent variables were sometimes not normally distributed.

To investigate emotion-specific differences in categorical congruence rates and compassionate empathy levels between patients and controls, independent samples t-tests were performed for cognitive and affective empathy, as well as for the motivational and ability components of compassionate empathy. For the exploratory analyses, no Bonferroni correction was applied in order to avoid a reduction in sensitivity.

## Supplementary Information

Below is the link to the electronic supplementary material.

Supplementary Material 1

## Data Availability

The data that support the findings of this study are available from the corresponding author upon reasonable request.
