# Supplementary material for: Reduced categorical congruence of cognitive and affective empathy in persons with psychotic disorders
Source: Sci Rep. 2026 Jan 14;16:1955. doi: 10.1038/s41598-025-34560-9 (PMC12804949; doi:10.1038/s41598-025-34560-9)
Supplement: Supplementary file 1 — Supplementary Material 1 [file 41598_2025_34560_MOESM1_ESM.docx]

**Reduced categorical congruence of cognitive and affective empathy**

**in persons with psychotic disorders**

Morini S.^*^, Hölz L., Kimmig A-C.S., Derntl B., Wildgruber D.

**Supplementary Information**

**1. Supplementary Methods**

***The Textual Empathy Test***

As the primary component of the experiment, all participants underwent the Textual Empathy Test, an empathy task adapted from previous studies[1-3]. The online questionnaire was generated using the software SoSci Survey[4] and was completed via the website [www.soscisurvey.de](http://www.soscisurvey.de/). The experiment was conducted in a quiet setting on a portable laptop with a stable internet connection. The duration of the test varied among participants, ranging from approximately 25 to 45 minutes.

This test involved the presentation of textual descriptions of 30 different real-life scenarios, each associated to a specific emotion chosen from a set of five positive (happiness, pride, gratitude, hope, and sexual arousal) and five negative emotions (anger, sadness, fear, shame, and disgust). Three scenarios were described that differed in the intensity of the respective emotion (low, medium, and high) for each of the ten emotional categories. The intensity levels differed significantly from each other in terms of mean valence (ranging from very unpleasant to very pleasant) and arousal ratings obtained in a preliminary study. Moreover, only scenarios which showed a correct categorical identification rate greater than 70% under forced choice conditions (selection from 10 emotional categories) were selected for the final test. Furthermore, it was confirmed that positive and negative emotions did not show significant differences in mean arousal and mean emotional intensity ratings (unpublished data from the pre-study). Table S1 provides an overview of the scenarios arranged in descending order of intensity (1: high intensity, 2: medium intensity, 3: low intensity).

Each situation was presented from the perspective of an unknown person of the participant's chosen gender (male or female). Each situation appeared on a separate page, arranged in a randomized order. At the beginning of every page of the questionnaire, participants were requested to read the scenario and imagine the unknown person, as described in the sentence introducing the scenario, experiencing the situation. Illustrative examples of the real-life scenarios presented in the test include the following (translated from the original German): “I'm going to the concert of a famous musician” (associated with happiness), “On my way home late at night, I hear fast footsteps behind me” (associated with fear), “Mr./Ms. Unknown is reading an erotic scene in a book” (associated with sexual arousal).

Participants were then asked to assign:

- the emotional state experienced by the unknown person (Figure S1)
- the emotional state experienced by themselves when the unknown person is in the situation

to one of ten categories (happiness, sexual arousal, hope, gratitude, pride, anger, fear, sadness, disgust, or shame) under forced-choice conditions.


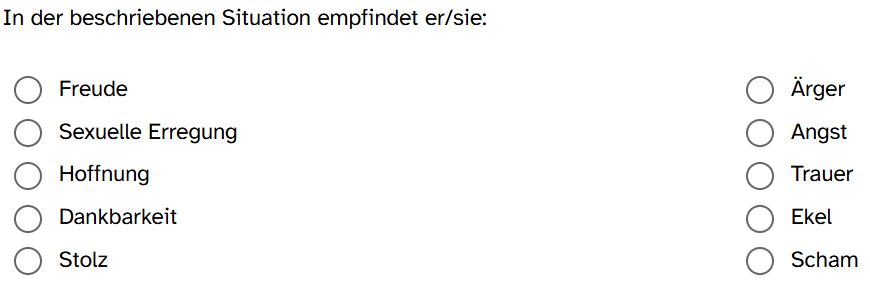


**Figure S1.** Illustrative example of a multiple-choice categorical emotion assignment for cognitive empathy (translated from the German: “In the described situation, he/she feels: (from top left to bottom right) happiness, sexual arousal, hope, gratitude, pride, anger, fear, sadness, disgust, shame”).

Moreover, on the same page, participants were requested to rate on two visual analogue scales:

- the intensity of their desire to provide emotional support to the unknown person (compassionate empathy – motivation) on the scale presented in Figure S2 (top)
- a self-estimation of their ability to emotionally support the unknown person (compassionate empathy – ability) on the scale presented in Figure S2 (bottom).


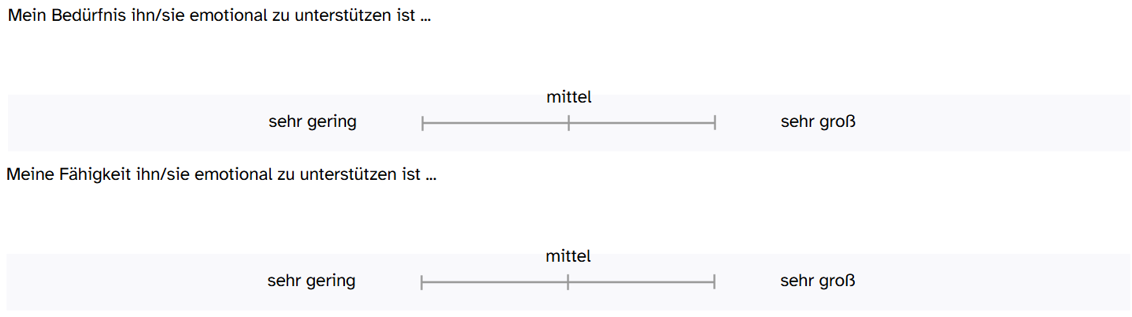


**Figure S2.** Illustrative example of visual analogue scale for compassionate empathy components (translated from the German: “My motivation (top) / ability (bottom) to support him/her emotionally is: very low – medium – very high”).

In summary, on one page, participants had to provide four answers.

Notably, in contrast to earlier versions of the TET[1-3], the scene descriptions remain visible on the screen until they actively click on them to move on to the next situation and the associated questions.

**Table S1.** Overview of the emotional scenarios included in the TET (adapted from[3]) in descending order of intensity (1: high intensity, 2: medium intensity, 3: low intensity).

| TET Stimuli for the unknown target person |
| --- |
| Positive Emotions |
| Joy   1. Frau/Herr Unbekannt erhält beim Lotto den Hauptgewinn.   *Ms./Mr. Unknown receives the main prize at the Powerball.*   1. Frau/Herr Unbekannts Lieblingslied kommt im Radio.   *Ms./Mr. Unknown’s favourite song is playing on the radio.*   1. Frau/Herr Unbekannt geht auf das Konzert eines bekannten Musikers.   *Ms./Mr. Unknown goes to the concert of a famous musician.* |
| Hope   1. Bei der Lottoziehung hat Frau/Herr Unbekannt die ersten Zahlen schon mal richtig.   *At the Powerball drawing, Ms./Mr. Unknown already has the first numbers correct.*   1. Frau/Herr Unbekannt hat die Möglichkeit, bei einem Preisausschreiben eine Reise zu gewinnen.   *Ms./Mr. Unknown has the opportunity to win a journey in a sweepstake.*   1. Frau/Herr Unbekannt ist schwer krank und beginnt eine vielversprechende Therapie.   *Ms./Mr. Unknown is seriously ill and is starting a promising therapy.* |
| Sexual Pleasure   1. Mit jeder weiteren Berührung wird ihr/sein Körper erregter.   *With each additional touch, her/his body becomes more aroused.*   1. Frau/Herrn Unbekannts Körper wird durch eine Berührung erregt.   *Ms./Mr. Unknown's body is aroused by a caresse*.   1. Frau/Herr Unbekannt liest eine erotische Szene in einem Buch.   *Ms./Mr. Unknown reads an erotic scene in a book.* |
| Gratefulness   1. Frau/Herr Unbekannt läuft mit Krücken, jemand hält die Tür auf.   *Ms./Mr. Unknown is walking with crutches, someone holds the door open.*   1. Frau/Herr Unbekannt liegt krank im Bett, jemand besorgt Medikamente.   *Ms./Mr. Unknown is sick in bed, someone buys medication.*   1. Jemand macht Frau/Herrn Unbekannt darauf aufmerksam, dass sie/er ein Handy liegen gelassen hat.   *Someone points out to Ms./Mr. Unknown that they left their phone behind.* |
| Pride   1. Frau/Herr Unbekannt hat eine schwierige Ausbildung erfolgreich abgeschlossen.   *Ms./Mr. Unknown has successfully completed a difficult training program.*   1. Frau/Herr Unbekannt schafft es eine lange Joggingrunde durchzuhalten.   *Ms./Mr. Unknown manages to complete a long jogging route.*   1. Frau/Herr Unbekannt hat eine Fastenzeit durchgehalten.   *Ms./Mr. Unknown completed a fasting period.* |
| Negative Emotions |
| Anger   1. Ihr/sein Geldbeutel wird auf einer belebten Straße gestohlen.   *Her/his wallet is stolen on a busy street.*   1. Die Bahn hat Verspätung, so dass Frau/Herr Unbekannt den Anschluss verpasst.   *The train is delayed, causing Ms./Mr. Unknown to miss her/his connection.*   1. Jemand hat Frau/Herrn Unbekannts Auto zugeparkt.   *Someone has double-parked next to Ms./Mr. Unknown's car.* |
| Disgust   1. Frau/Herr Unbekannt muss sich auf eine verschmutzte Toilette setzen.   *Ms./Mr. Unknown has to sit on a dirty toilet.*   1. Frau/Herr Unbekannt entdeckt Maden im Biomüll.   *Ms./Mr. Unknown finds maggots in the organic waste.*   1. Frau/Herr Unbekannt sieht Erbrochenes auf der Straße.   *Ms./Mr. Unknown sees vomit on the street.* |
| Fear   1. Frau/Herr Unbekannt wacht auf und sieht Flammen in der Wohnung.   *Ms./Mr. Unknown wakes up and sees flames in the apartment.*   1. Nachts auf dem Heimweg bemerkt Frau/Herr Unbekannt schnelle Schritte hinter sich.   *At night on the way home, Ms./Mr. Unknown notices fast footsteps behind her/him.*   1. Ein bedrohlicher Hund kommt auf Frau/Herrn Unbekannt zu gerannt.   *A dangerous dog comes running toward Ms./Mr. Unknown.* |
| Sadness   1. Frau/Herr Unbekannt erfährt vom Tod einer Person, die sie/er sehr gerne gehabt hat.   *Ms./Mr. Unknown learns of the death of a person she/he was very fond of.*   1. Frau/Herrn Unbekannts Katze muss auf Grund einer Krankheit eingeschläfert werden.   *Ms./Mr. Unknown’s cat has to be put down due to an illness.*   1. Es ist der Todestag von Frau/Herrn Unbekannts Mutter.   *It is the anniversary of Ms./Mr. Unknown's mother's death.* |
| Shame |
| 1. Frau/Herr Unbekannt wird von den Eltern beim Sex erwischt.   *Ms./Mr. Unknown gets caught by her/his parents having sex.*   1. Frau/Herr Unbekannt bückt sich in der Öffentlichkeit, die Hose reißt am Po.   *Ms./Mr. Unknown bends down in public, and her/his pants tear at the back.*   1. Frau/Herr Unbekannt winkt jemandem freundlich "zurück" und bemerkt, dass sie/er gar nicht gemeint war.   *Ms./Mr. Unknown waves back to someone and realizes that she/he wasn't the person intended.* |

**2. Supplementary Analyses**

***2.1 Emotion-specific differences in categorical congruence rates of cognitive empathy and identification accuracy from the self-perspective in healthy participants.***

In the present study, the concept of categorical congruence was operationalized as the agreement between the category of the emotion intended to be evoked by the textual description and the participants' assessment of another person's emotion in the given situation (cognitive empathy) or their emotion they felt themselves when the other person is experiencing the situation (affective empathy). It should be noted, however, that the preliminary study conducted to select situation descriptions unequivocally assigned to specific emotional categories showed differences in identification accuracy between specific emotions.

In the preliminary study, a different sample of 23 healthy participants were asked to imagine experiencing the described situation themselves and to assign their emotional response to one of the ten emotional categories under forced-choice conditions. The categorical congruence rate of the responses achieved from the self-perspective with the emotion intended in the scenario averaged to 92.5% and was virtually identical to the categorical congruence rate of cognitive empathic responses in the healthy control group in the current study (92.7%). The emotion-specific categorical congruence rates of cognitive empathic responses in the present study were compared with congruence rates in the self-perspective obtained in the preliminary study using t-tests for independent samples. The results showed no significant differences in congruence rates for any emotional category (see Table S2).

***Table S2.*** *Emotion-specific categorical congruence rates of cognitive empathy (present study) and self-perspective (preliminary study, carried out with 23 different participants) in healthy participants. M = mean value; SD = standard deviation.*

|  | **Cognitive Empathy**  **(present study, n=21)** | **Self-Perspective**  **(pre-study, n=23)** | **Cognitive Empathy vs. Self Perspective** | |
| --- | --- | --- | --- | --- |
|  | M ± SD | M ± SD | T | p |
| **Anger** | 98.4 ± 7.3 | 98.6 ± 7.0 | -0.11 | 0.96 |
| **Fear** | 98.4 ± 7.3 | 98.6 ± 67.0 | -0.11 | 0.95 |
| **Sadness** | 100.0 ± 0.0 | 95.7 ± 15.3 | 1.31 | 0.20 |
| **Disgust** | 95.2 ± 15.9 | 97.1 ± 9.6 | -0.55 | 0.64 |
| **Shame** | 93.8 ± 22.8 | 89.9 ± 25.5 | 0.52 | 0.60 |
| **Happiness** | 93.8 ± 13.4 | 95.7 ± 11.5 | -0.53 | 0.61 |
| **Sexual arousal** | 96.8 ± 14.7 | 92.75 ± 14.1 | 0.94 | 0.35 |
| **Hope** | 84.1 ± 22.8 | 73.9 ± 28.4 | 1.31 | 0.21 |
| **Gratitude** | 85.7 ± 22.5 | 94.2 ± 16.4 | -1.44 | 0.17 |
| **Pride** | 81.0 ± 27.0 | 88.4 ± 16.2 | -1.12 | 0.28 |
| **Total** | 92.7 ± 7.9 | 92.5 ± 5.8 | 0.11 | 0.91 |

***2.2 Emotion-specific differences in categorical congruence rates***

Using paired samples t-tests, categorical congruence rates for each emotion were compared to the average values of the remaining nine emotions within each group. Moreover, emotion-specific mean values were compared between patients with psychotic disorders and healthy controls. These emotion-specific results are presented in Tables S3-S4. For the exploratory analyses, no Bonferroni correction was applied in order to avoid a reduction in sensitivity.

***Table S3.*** ***Comparison of the categorical congruence of cognitive empathy among different emotions and groups****. HC: healthy control, M = mean value; SD = standard deviation. The T- and two-sided p-values in the first two columns refer to the comparison of the categorical congruence rate for the specific emotion with the rates of the other nine emotions. p-values indicating significant results are highlighted in bold. *p < 0.05, **p < 0.01, ***p < 0.001*

| Emotion | **Healthy Control** | | | **Psychotic Disorder** | | | **Psychotic Disorder vs. HC** | | |
| --- | --- | --- | --- | --- | --- | --- | --- | --- | --- |
|  | M ± SD | T | p | M ± SD | T | p | M ± SD | T | p |
| **Anger** | 98.4 ± 7.3 | 2.71 | **0.01*** | 85.7 ± 22.5 | 1.36 | 0.19 | -12.7 ± 5.2 | 2.46 | **0.02*** |
| **Fear** | 98.4 ± 7.3 | 2.71 | **0.01*** | 98.4 ± 7.3 | 6.28 | **<0.001***** | 0.0 ± 2.2 | 0.00 | 1.00 |
| **Sadness** | 100.0 ± 0.0 | 4.30 | **<0.001***** | 96.8 ± 10.0 | 5.36 | **<0.001***** | -3.2 ± 2.2 | 1.45 | 0.16 |
| **Disgust** | 95.2 ± 15.9 | 0.97 | 0.34 | 92.1 ± 14.6 | 4.66 | **<0.001***** | -3.2 ± 4.7 | 0.67 | 0.50 |
| **Shame** | 93.7 ± 22.7 | 0.23 | 0.82 | 73.0 ± 40.3 | -1.06 | 0.30 | -20.6 ± 10.1 | 2.05 | **0.05*** |
| **Happiness** | 93.7 ± 13.4 | 0.31 | 0.76 | 92.1 ± 14.6 | 2.71 | **0.01*** | -1.6 ± 4.3 | 0.37 | 0.72 |
| **Sexual arousal** | 96.8 ± 14.6 | 1.38 | 0.18 | 85.7 ± 22.5 | 1.05 | 0.31 | -11.1 ± 5.9 | 1.90 | 0.07 |
| **Hope** | 84.1 ± 22.7 | -2.10 | **0.05*** | 61.9 ± 33.8 | -3.22 | **<0.01**** | -22.2 ± 8.9 | 2.50 | **0.02*** |
| **Gratitude** | 85.7 ± 22.6 | -1.73 | 0.10 | 74.6 ± 23.3 | -1.73 | 0.10 | -11.1 ± 7.1 | 1.57 | 0.12 |
| **Pride** | 81.0 ± 27.0 | -2.25 | **0.04*** | 49.2 ± 32.7 | -5.14 | **<0.001***** | -31.8 ± 9.3 | 3.43 | **<0.01**** |
| **Total** | 92.7 ± 7.8 | - | - | 81.0 ± 12.1 | - | - | -11.8 ± 3.1 | 3.75 | <**0.001***** |

**Table S4. Comparison of the categorical congruence of affective empathy among different emotions and groups**. HC: healthy control, M = mean value; SD = standard deviation. The T- and two-sided p-values in the first two columns refer to the comparison of the categorical congruence rate for the specific emotion with the rates of the other nine emotions. p-values indicating significant results are highlighted in bold within the respective tables. *p < 0.05, **p < 0.01, ***p < 0.001

| Emotion | **Healthy Control** | | | **Psychotic Disorder** | | | **Psychotic Disorder vs. HC** | | |
| --- | --- | --- | --- | --- | --- | --- | --- | --- | --- |
|  | M ± SD | T | p | M ± SD | T | p | M ± SD | T | p |
| **Anger** | 84.1 ± 31.0 | 3.07 | **<0.01**** | 57.1 ± 35.2 | 1.03 | 0.32 | -27.0 ± 10.2 | 2.64 | **0.01*** |
| **Fear** | 82.5 ± 31.0 | 2.83 | **0.01*** | 71.4 ± 39.8 | 3.44 | **<0.01**** | -11.1 ± 11.0 | 1.01 | 0.32 |
| **Sadness** | 90.5 ± 6.1 | 4.94 | **<0.001***** | 73.0 ± 34.4 | 4.31 | **<0.001***** | -17.5 ± 9.4 | 1.85 | 0.07 |
| **Disgust** | 84.1 ± 27.1 | 4.05 | **<0.001***** | 76.2 ± 28.2 | 6.33 | **<0.001***** | -7.9 ± 8.5 | 0.93 | 0.36 |
| **Shame** | 66.7 ± 36.5 | 0.23 | 0.82 | 47.6 ± 37.4 | -0.48 | 0.64 | -19.1 ± 11.4 | 1.67 | 0.10 |
| **Happiness** | 93.7 ± 13.4 | 9.05 | **<0.001***** | 79.4 ± 2.3 | 6.33 | **<0.001***** | -14.3 ± 5.7 | 2.52 | **0.02*** |
| **Sexual arousal** | 33.3 ± 39.4 | -3.55 | **<0.01**** | 33.3 ± 42.2 | -1.81 | 0.09 | 0.0 ± 12.6 | 0.00 | 1.00 |
| **Hope** | 60.3 ± 34.4 | -0.69 | 0.50 | 28.6 ± 28.5 | -4.07 | **<0.001***** | -31.8 ± 9.7 | 3.26 | **<0.01**** |
| **Gratitude** | 28.6 ± 30.3 | -6.22 | **<0.001***** | 28.6 ± 21.8 | -3.97 | **<0.001***** | 0.0 ± 8.2 | 0.00 | 1.00 |
| **Pride** | 27.0 ± 34.4 | -6.18 | **<0.001***** | 15.9 ± 22.7 | -7.41 | **<0.001***** | -11.1 ± 9.0 | 1.24 | 0.22 |
| **Total** | 65.1 ± 12.8 | - | - | 51.1 ± 16.1 | - | - | -14.0 ± 4.5 | 3.12 | **<0.01**** |

***2.3 Emotion-specific differences in compassionate empathy levels***

Using paired samples t-tests, compassionate empathy levels for each emotion were compared to the average values of the remaining nine emotions within each group. Moreover, emotion-specific mean values were compared between patients with psychotic disorders and healthy controls. These emotion-specific results are presented in Tables S5-S6. For the exploratory analyses, no Bonferroni correction was applied in order to avoid a reduction in sensitivity.

***Table S5. Comparison of compassionate empathy (motivation) levels among different emotions and groups.*** *HC: healthy control, M = mean value; SD = standard deviation. The t- and two-sided p-values in the first two columns refer to the comparison of the compassionate empathy level for the specific emotion with the levels of the other nine emotions. p-values indicating significant results are highlighted in bold within the respective tables. *p < 0.05, **p < 0.01, ***p < 0.001*

| Emotion | **Healthy Control** | | | **Psychotic Disorder** | | | **Psychotic Disorder vs. HC** | | |
| --- | --- | --- | --- | --- | --- | --- | --- | --- | --- |
|  | M ± SD | T | p | M ± SD | T | p | M ± SD | T | p |
| **Anger** | 67.2 ± 15.3 | 9.10 | **<0.001***** | 67.6 ± 15.0 | 4.83 | **<0.001***** | +0.4 ± 4.7 | -0.08 | 0.94 |
| **Fear** | 64.9 ± 18.8 | 5.71 | **<0.001***** | 69.9 ± 19.1 | 5.81 | **<0.001***** | +5.0 ± 5.8 | -0.85 | 0.40 |
| **Sadness** | 51.3 ± 10.4 | 0.67 | 0.51 | 47.5 ± 12.7 | 1.71 | 0.10 | -3.8 ± 3.6 | 1.05 | 0.30 |
| **Disgust** | 41.8 ± 18.2 | -3.87 | **<0.001***** | 46.2 ± 24.9 | -2.60 | **0.02*** | +4.4 ± 6.7 | -0.65 | 0.52 |
| **Shame** | 43.6 ± 19.6 | -2.22 | **0.04*** | 47.1 ± 21.6 | -2.53 | **0.02*** | +3.5 ± 6.3 | -0.55 | 0.59 |
| **Happiness** | 51.7 ± 15.5 | 1.24 | 0.23 | 58.3 ± 21.2 | 1.05 | 0.31 | +6.6 ± 5.7 | -1.14 | 0.26 |
| **Sexual arousal** | 36.8 ± 19.0 | -4.65 | **<0.001***** | 47.5 ± 29.4 | -1.42 | 0.17 | +10.7 ± 7.6 | -1.41 | 0.17 |
| **Hope** | 38.7 ± 19.9 | -3.98 | **<0.001***** | 41.5 ±28.2 | -3.66 | **<0.01**** | +2.8 ± 7.5 | -0.37 | 0.71 |
| **Gratitude** | 41.6 ± 22.5 | -2.22 | **0.04*** | 55.2 ± 25.4 | 0.18 | 0.86 | +13.6 ± 7.4 | -1.83 | 0.08 |
| **Pride** | 60.0 ± 17.3 | 3.48 | **<0.01**** | 64.7 ± 20.6 | 4.57 | **<0.001***** | +4.7 ± 5.9 | -0.80 | 0.43 |
| **Total** | 51.4 ± 13.7 | - | - | 56.4 ± 16.7 | - | - | +5.0 ± 4.7 | -1.06 | 0.29 |

***Table S6. Comparison of compassionate empathy (ability) levels among different emotions and groups.*** *HC: healthy control, M = mean value; SD = standard deviation. The t- and two-sided p-values in the first two columns refer to the comparison of the compassionate empathy level for the specific emotion with the levels of the other nine emotions. p-values indicating significant results are highlighted in bold within the respective tables. *p < 0.05, **p < 0.01, ***p < 0.001*

| Emotion | **Healthy Control** | | | **Psychotic Disorder** | | | **Psychotic Disorder vs. HC** | | |
| --- | --- | --- | --- | --- | --- | --- | --- | --- | --- |
|  | M ± SD | T | p | M ± SD | T | p | M ± SD | T | p |
| **Anger** | 57.4 ± 17.1 | 2.92 | **<0.01**** | 59.3 ± 24.0 | 0.62 | 0.54 | +1.9 ± 6.4 | -0.30 | 0.77 |
| **Fear** | 62.8 ± 21.9 | 4.21 | **<0.001***** | 67.6 ± 25.4 | 3.29 | **<0.01**** | +4.9 ± 7.3 | -0.67 | 0.51 |
| **Sadness** | 59.6 ± 17.1 | 2.90 | **<0.01**** | 63.1 ± 22.1 | 1.59 | 0.12 | +3.5 ± 6.1 | -0.57 | 0.57 |
| **Disgust** | 40.8 ± 24.4 | -2.92 | **<0.01**** | 47.5 ± 26.0 | -3.32 | **<0.01**** | +6.6 ± 7.8 | -0.85 | 0.40 |
| **Shame** | 48.5 ± 24.2 | -0.67 | 0.51 | 52.6 ± 20.9 | -2.49 | **0.02*** | +4.0 ± 7.0 | -0.58 | 0.57 |
| **Happiness** | 47.6 ± 23.2 | -0.96 | 0.35 | 56.6 ± 22.5 | -0.31 | 0.76 | +9.1 ± 7.1 | -1.29 | 0.21 |
| **Sexual arousal** | 28.0 ± 29.0 | -5.48 | **<0.001***** | 46.6 ± 34.5 | -2.18 | **0.04*** | +18.6 ± 9.8 | -1.90 | 0.07 |
| **Hope** | 50.0 ± 20.3 | -0.10 | 0.92 | 58.6 ± 22.5 | 0.51 | 0.61 | +8.5 ± 6.6 | -1.29 | 0.20 |
| **Gratitude** | 46.5 ± 25.0 | -1.25 | 0.23 | 59.8 ± 26.3 | 0.96 | 0.35 | +13.3 ± 7.9 | -1.68 | 0.10 |
| **Pride** | 61.5 ± 19.7 | 3.41 | **<0.01**** | 63.3 ± 22.7 | 2.15 | **0.04*** | +1.9 ± 6.6 | -0.29 | 0.78 |
| **Total** | 50.3 ± 17.7 | - | - | 57.5 ± 20.6 | - | - | +7.2 ± 5.9 | -1.22 | 0.23 |

***2.4 Emotion-specific differences in incongruent categorical attributions***

Using independent samples t-tests, emotion-specific categorical attribution rates for both cognitive and affective empathy were compared between patients with psychotic disorders and healthy controls. The results of these analyses are shown in Figure S3.

| 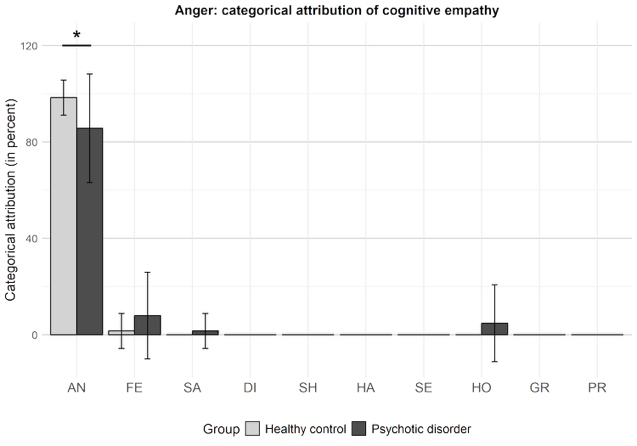 | 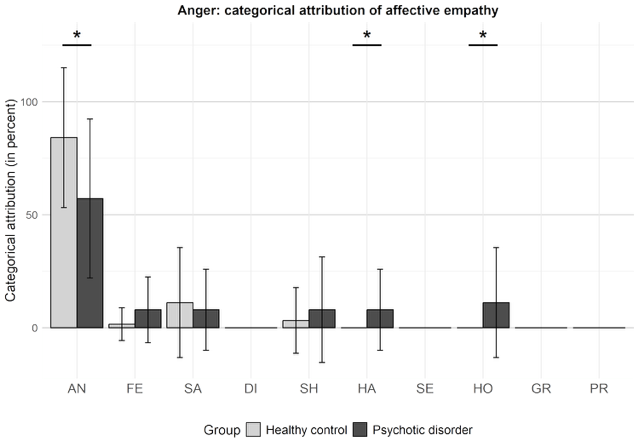 |
| --- | --- |
| 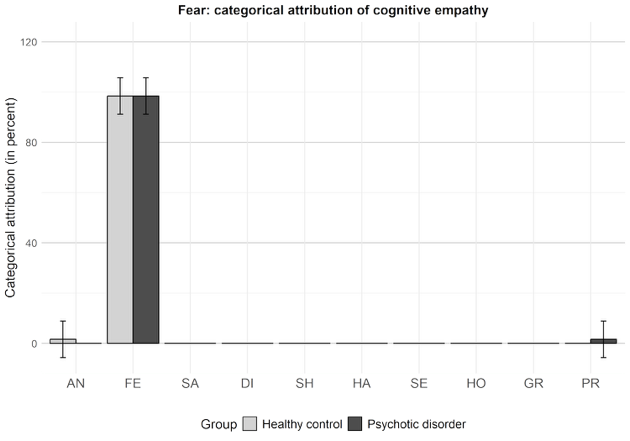 | 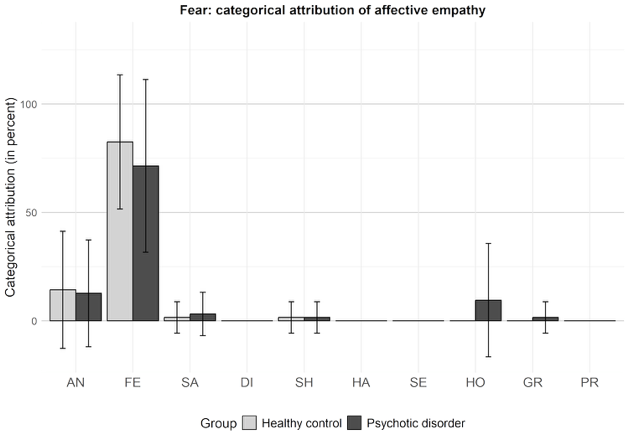 |
| 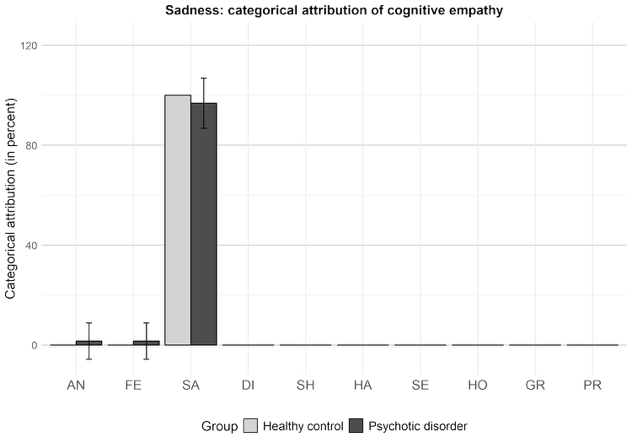 | 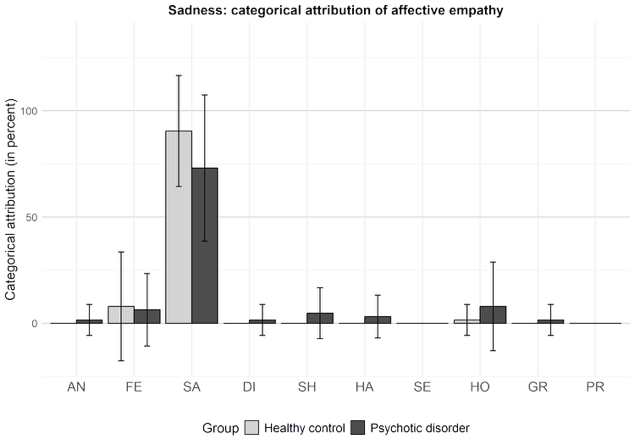 |
| 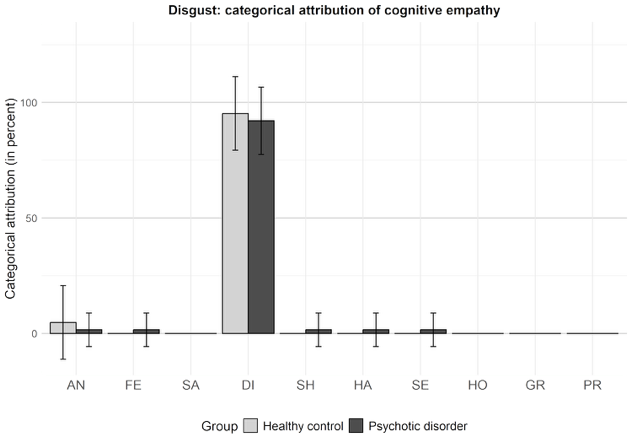 | 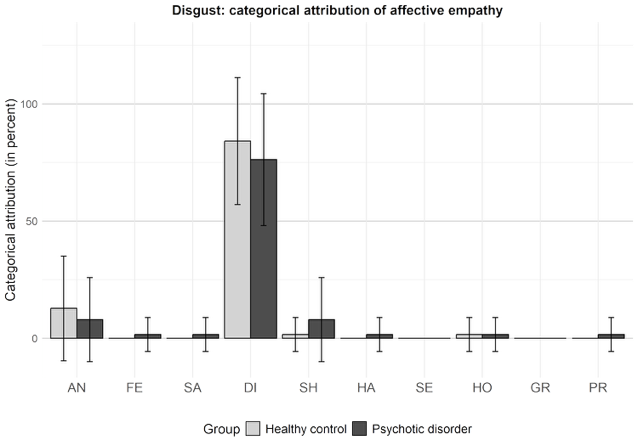 |
| 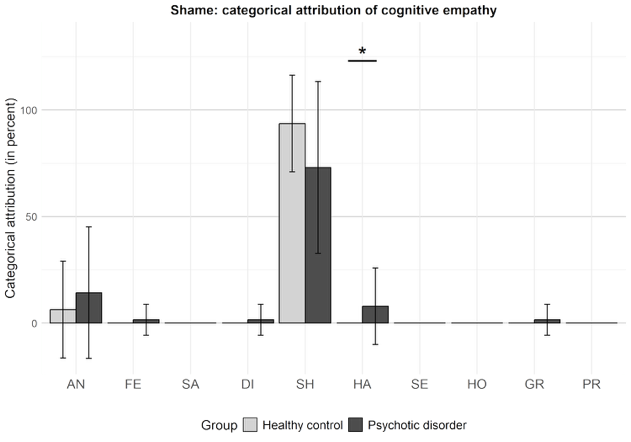 | 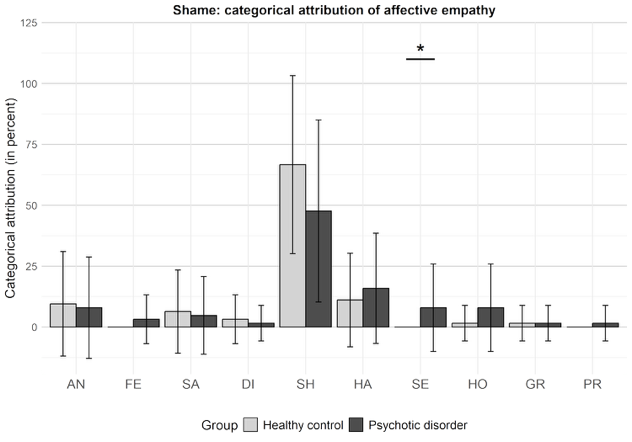 |
| 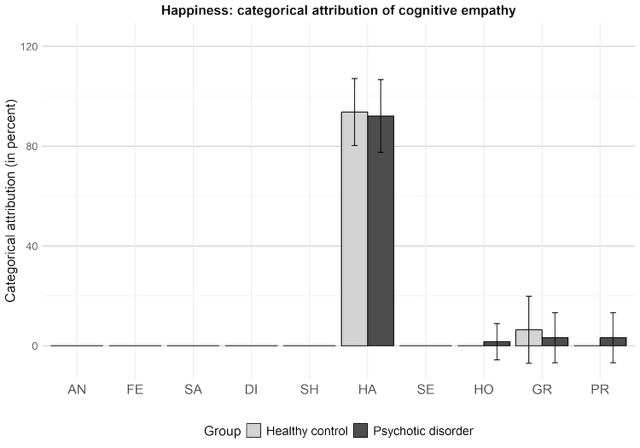 | 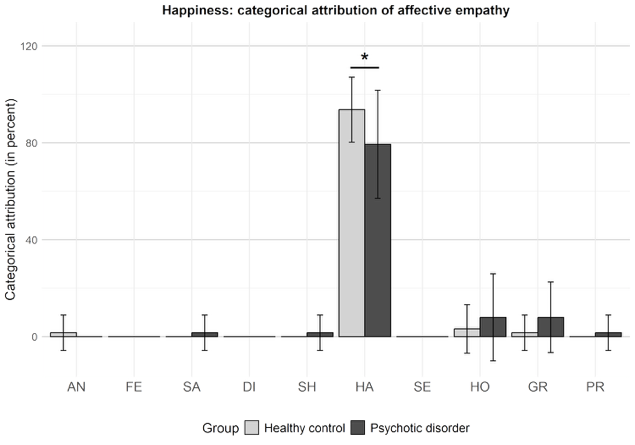 |
| 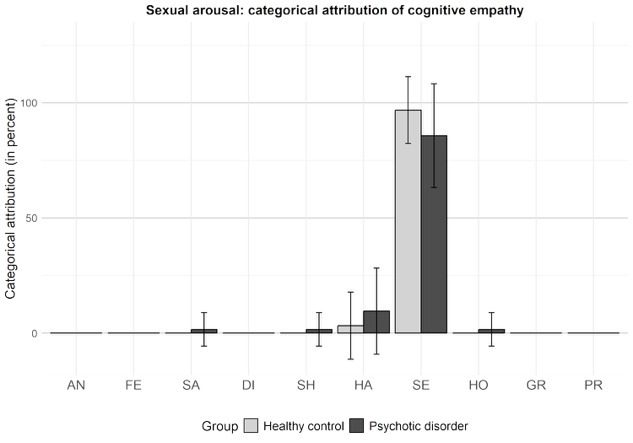 | 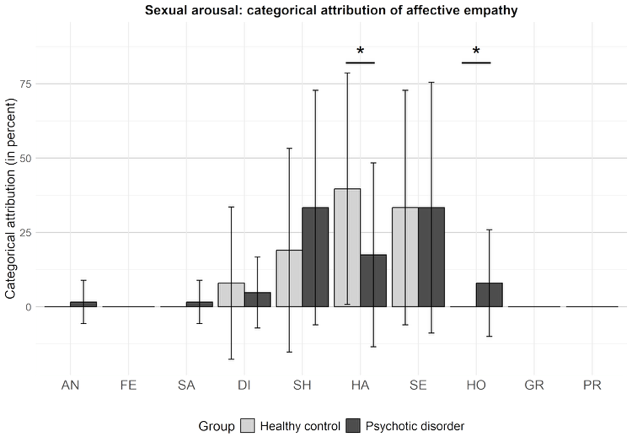 |
| 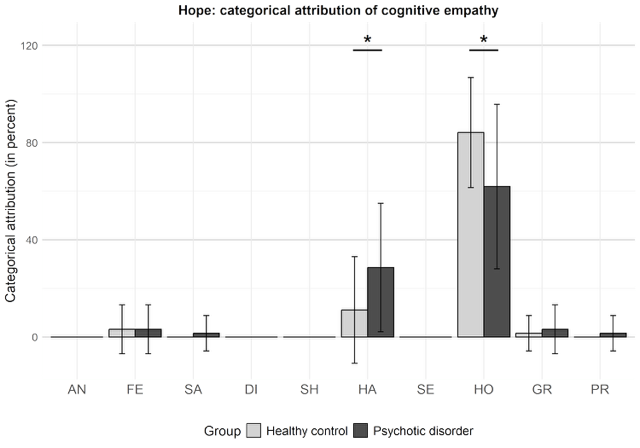 | 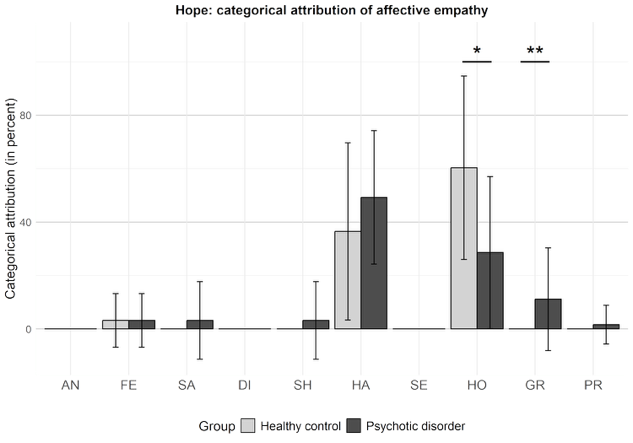 |
| 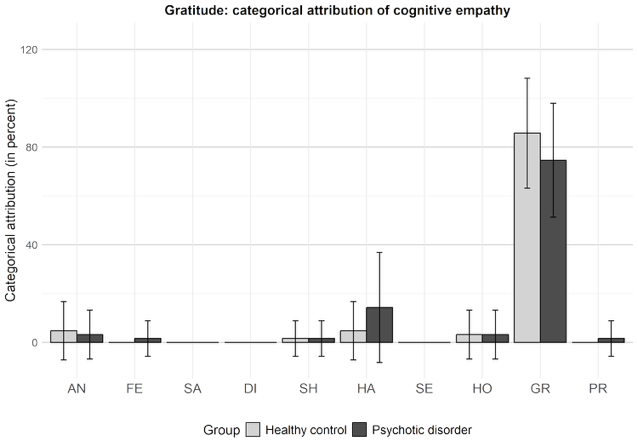 | 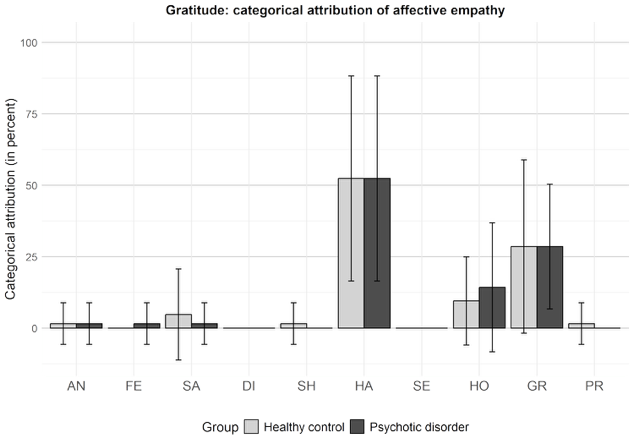 |
| 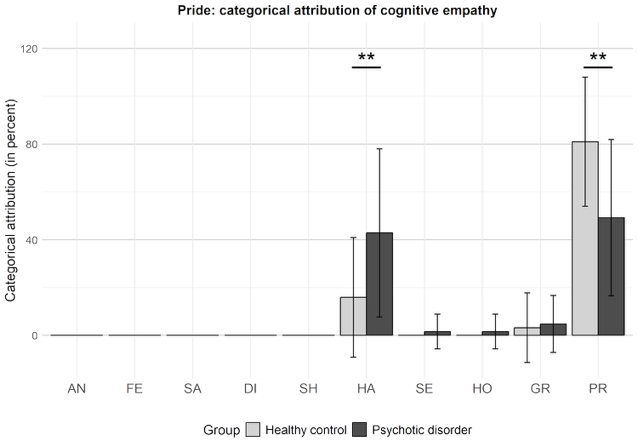 | 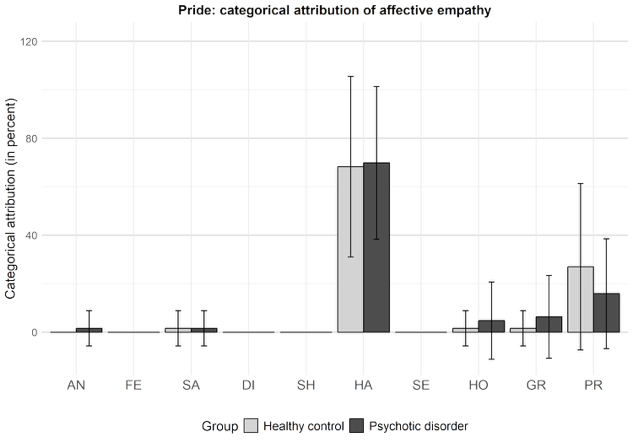 |

**Figure S3**. **Emotion-specific categorical attribution rates for cognitive and affective empathy in patients and controls.** The columns represent the mean categorical attribution rate of cognitive (figures on the left) or affective (figures on the right) empathy for controls (light grey) and patients with psychotic disorder (dark grey). The error bars indicate the respective standard deviations. On the X-axis: AN: anger, FE: fear, SA: sadness, DI: disgust, SH: shame, HA: happiness, SE: sexual arousal, HO: hope, GR: gratitude, PR: pride. *p < 0.05, **p < 0.01, ***p < 0.001

Group differences in incongruent categorical attributions arose for the emotions anger, shame, happiness, sexual arousal, hope, and pride. The remaining four emotions did not show any group differences in categorical attributions.

Regarding the emotions anger and shame, the responses given by patients with psychotic disorders instead of the intended emotion appear to be nonspecifically distributed across the alternative categories and can probably be attributed to deficits in emotion identification or to the fact that the range of possible responses was limited – so that in case of uncertainty or assumed absence of emotional responses, the participant may have made an arbitrary choice. In the case of happiness, hope and pride, the differences mainly consisted of a substitution with another positive emotion. This could be due to a limited ability to distinguish between two positive emotions (cognitive empathy, in the case of hope) or a general tendency to experience a positive emotion when observing a scene in which another person is feeling a positive emotion (affective empathy, in the case of happiness, hope and pride).

Finally, for stimuli with textual descriptions of sexual arousal, independent samples t-tests showed that patients chose happiness as an answer significantly less often than controls (Mean difference = -0.22, Standard error difference = 0.11, p = 0.047), whereas patients chose hope significantly more often (Mean difference = 0.08, Standard error difference= 0.04, p = 0.0496). Additionally, patients chose shame as an answer more often compared to controls, but this difference was not statistically significant (p = 0.22). A possible explanation is that individuals with psychotic disorders may have different experiences with sexuality compared to the control group, partly due to greater difficulties in finding a partner - resulting from mistrust as a feature of positive symptoms, social withdrawal as part of negative symptoms, impairments in social cognition, stigmatization, and sexual dysfunction as a side effect of antipsychotic medication[5, 6]. This could lead to a change in the person's affective empathy reaction when experiencing other people having sexual experiences (for instance by reacting with shame).

**Bibliography**

1. Kimmig, A.S., et al., *Friend vs. foe: Cognitive and affective empathy in women with different hormonal states.* Frontiers in Neuroscience, 2021. **15**: p. 608768.

2. Kimmig, A.S., et al., *Lower affective empathy in oral contraceptive users: a cross-sectional fMRI study.* Cerebral Cortex, 2023. **33**(8): p. 4319-4333.

3. Kimmig, A.S., et al., *Impairment of affective and cognitive empathy in high functioning autism is mediated by alterations in emotional reactivity.* Scientific reports, 2024. **14**(1): p. 21662.

4. Leiner, D.J., *SoSci Survey (Version 3.5.00)*. 2019.

5. Baggaley, M., *Sexual dysfunction in schizophrenia: focus on recent evidence.* Human Psychopharmacology, 2008. **23**(3): p. 201-9.

6. Budziszewska, M.D., M. Babiuch-Hall, and K. Wielebska, *Love and romantic relationships in the voices of patients who experience psychosis: an interpretive phenomenological analysis.* Frontiers in Psychology, 2020. **11**: p. 570928.
